# mSphere of Influence: Innate Immunity at the Maternal-Fetal Barrier

**DOI:** 10.1128/mSphere.00639-20

**Published:** 2020-07-15

**Authors:** Kellie Ann Jurado

**Affiliations:** a Department of Microbiology, University of Pennsylvania, Philadelphia, Pennsylvania, USA

**Keywords:** innate immunity, placental immunology, reproductive immunology

## Abstract

Kellie Ann Jurado works in the field of emerging infectious diseases. In this mSphere of Influence article, she reflects on how the papers “Type III interferons produced by human placental trophoblasts confer protection against Zika virus infection” (https://doi.org/10.1016/j.chom.2016.03.008) and “A three-dimensional culture system recapitulates placental syncytiotrophoblast development and microbial resistance” (https://doi.org/10.1126/sciadv.1501462) by Carolyn Coyne’s group have made an impact on her, inspiring her to explore immunity in the placenta by indicating the unique innate immune control elicited at the maternal-fetal barrier as well as by providing physiologically relevant model systems for study.

## COMMENTARY

The placenta protects the developing fetus from maternal threats, including infectious pathogens, by embodying various barrier functions, making the maternal-fetal interface typically refractory to viral infection. Carolyn Coyne’s group at the University of Pittsburgh has provided fundamental insight into the different immunological protections used by the placenta to protect the fetus from congenital infection. Further, her group has established novel, genetically manipulatable, and physiologically relevant platforms that effectively model the human maternal-fetal interface and allow for interrogation of mechanisms involved in immunological protection of placental microbial resistance.

Maternal and fetal blood are separated by a cellular barrier comprised of a network of fused cytotrophoblast cells termed syncytiotrophoblasts. These cells serve as the front line of fetal defense. Historically, biomedical research aimed at defining women’s reproductive biology has been understudied, in part due to experimental limitations in the reproductive sciences that have made it difficult to comprehensively investigate pregnancy processes. This has necessitated innovative methods to model human reproduction in a physiologically relevant manner. “A three-dimensional culture system recapitulates placental syncytiotrophoblast development and microbial resistance” (1) was the first platform to provide reproductive immunologists with a genetically tractable system to investigate innate immunity of the placenta with a model that bears extraordinarily similar transcriptional profiles to those of primary human syncytiotrophoblasts. The authors achieve this platform by taking advantage of culturing cells in three dimensions (3D). The use of a rotating wall vessel bioreactor (originally developed by NASA) for the culture of cells creates an environment with a physiological level of fluid shear stress and mass transfer tending toward optimal cell growth and differentiation due to a lack of sedimentation. The manuscript describes how a coculture of a human choriocarcinoma cell line, JEG-3, with endothelial cells under these 3D conditions resulted in efficient syncytium formation, hormone production, and pathogen restriction of common congenital pathogens. Thereby, this platform opens the door to reproductive scientists to deep-dive into syncytiotrophoblast functions at large and also to uncover immunological protective mechanisms employed at the maternal-fetal interface.

Zika virus, a pathogen that mostly causes mild disease in healthy individuals, was found to instigate severe complications when congenitally contracted. This disease presentation quickly called into question how placental barriers were breached in order to ultimately result in fetal infection. The paper entitled “Type III interferons produced by human placental trophoblasts confer protection against Zika virus infection” by Bayer et al. (2) beautifully revealed the critical role of interferon lambda 1 (IFNλ1) in mediating antiviral defense at the maternal-fetal interface. The authors initially found that primary human trophoblasts (PHT) were unable to be infected by Zika virus and that priming of nonplacental cells with PHT-conditioned medium was able to initiate protective antiviral programs. This finding indicated that PHT were releasing a signal that induced an antiviral program. The authors identified the signal to be IFNλ1. Overall, the manuscript illustrates how IFNλ1, made and secreted from placental trophoblasts, is a major immunological protection mechanism of the placenta and is the reason this fundamental cellular barrier is able to effectively prohibit Zika virus infection.

Yet, in my opinion, the most intriguing part of this work was that the authors found IFNλ1 production to happen in the absence of an active viral infection. Thereby, under baseline conditions, IFNλ1 production provides the maternal-fetal interface with antiviral properties prior to detection of a viral infection. This finding highlights the placenta as an immunologically unique organ that has evolved distinct immune control mechanisms. Live bearing in mammals is critically dependent upon maternal immune tolerance of a genetically dissimilar fetus and tissue-invasive placenta. This process necessitates the induction and maintenance of an immunosuppressive environment to prevent maternally mediated immune rejection of offspring. Yet, this finding underscored the necessity of still employing an active antiviral defense in the case of maternal infection during pregnancy. This work has initiated a gnawing curiosity in me to further investigate immune control mechanisms that have evolved at this unique barrier interface and that underlie the evolutionarily essential balancing act of initiating effective immune defenses against congenital infections while also remaining tolerogenic to a genetically dissimilar fetus.

Since these field-altering articles have been published from the Coyne group, reproductive immunology as a whole has continued to flourish and generate important works that further feature the distinct and central role immune signaling plays at the maternal-fetal interface. Moreover, type III interferons have become more and more appreciated for nonredundant roles in mitigating local infection control at barrier regions. Although placental immunology has been long left understudied, the intricate details that evolutionary pressures have refined are finally being queried and appreciated.
